# Editor’s introduction

**DOI:** 10.1186/s41039-015-0021-1

**Published:** 2015-11-06

**Authors:** Siu-Cheung Kong

**Affiliations:** Department of Mathematics and Information Technology, The Hong Kong Institute of Education, 10 Lo Ping Road, Tai Po, New Territories, Hong Kong, China

In this 2015 third issue of *Research and Practice in Technology Enhanced Learning*, we are delighted to present a Special Issue on the theme “ICT in Language Learning” with three selected articles, on top of our regular publication of original articles.

The Special Issue on the theme “ICT in Language Learning”, with Yu-Ju Lan and Ting-Chia Hsu as Guest Editors, aims to disseminate the practical application of advanced ICT technology to language teaching and learning. The Editorial by the Guest Editors delineates the theme of the Special Issue as well as introduces the individual articles. I would like to take this opportunity to sincerely thank the Guest Editors for their continuous commitment and professional input in the realization of the Special Issue.

Apart from the three Special Issue Articles, this issue presents three Original Articles that focus on a ubiquitous computer system for acculturation in context, a framework of concept map for automatic assessment, and a monitoring system for programming practice in lectures.

In the paper *Acculturation in Context: Knowledge Sharing through Ubiquitous Technologies*, Cook et al. report on the development and evaluation of a platform which uses a retooled mobile and ubiquitous computer system to facilitate knowledge dissemination among foreign postgraduate students during the process of acculturation. Through a four-stage study adopting both quantitative and qualitative methods, the authors identify measurable benefits that the designed platform brings about to the target students in the process of acculturation. The authors also discuss three directions to expand the current work on using ubiquitous computing and mobile technology for acculturation.

In the paper *Framework of Kit-Build Concept Map for Automatic Diagnosis and its Preliminary Use*, Hirashima et al. propose a framework of Kit-Build Concept Map (KB map) as a promising method to realize automatic assessment of a concept map. The “KB map System” is developed as a web application with three client systems for students to create goal maps and group maps. The preliminary use of such system by undergraduate students confirms the feasibility of using the four-phase framework with the designed system for supporting students to detect areas of improvement in subject learning. The authors also discuss their future work on expanding the use of the designed system with the proposed framework across more student grades and subject areas.

In the paper *Monitoring System for the Effective Instruction based on the Semi-Automatic Evaluation of Programs During Programming Classroom Lectures*, Kogure et al. report on the development of a programming practice monitoring system to facilitate teachers to give appropriate instructions to students at the right time during classroom lectures. The monitoring system is designed with five functions for an automatic collection and analysis of programs written by students. Through an experimental evaluation, the authors confirm the usefulness of the designed system with five functions for supporting programming instruction in real classroom settings, and its high accuracy in evaluating student programs.

We keep soliciting an eclectic collection of quality paper submissions from researchers and practitioners around the world to share insights into the theoretical and methodological dimensions of research and practice in technology enhanced learning.

*KONG, Siu Cheung*

Editor-in-Chief

